# Immune checkpoint inhibitor-related pneumonitis: current advances and the putative role of mesenchymal stem cell therapy

**DOI:** 10.1038/s41419-026-08440-7

**Published:** 2026-02-03

**Authors:** Zekang Li, Xiao Zheng, Han Xia, Long Lu, Xiaodong Chen, Yongjing Chen, Jun Wu, Yufang Shi, Chen Wu

**Affiliations:** 1 Department of Oncology, The Third Affiliated Hospital of Soochow University, Changzhou, Jiangsu China; 2 Department of Biotherapy, The Third Affiliated Hospital of Soochow University, Changzhou, Jiangsu China; 3 Institute of Cell Therapy, Soochow University, Changzhou, Jiangsu China; 4 Department of Oncology, The First People’s Hospital of Changzhou, Changzhou, Jiangsu China; 5 Department of Tumor Biological Treatment, The Third Affiliated Hospital of Soochow University, Changzhou, Jiangsu China; 6 Jiangsu Engineering Research Center for Tumor Immunotherapy, Changzhou, Jiangsu China; 7 Department of Oncology, The First People’s Hospital of Jingzhou, Jingzhou, Hubei China; 8 Wuxi Sinotide New Drug Discovery Institutes, Huishan Economic and Technological Development Zone, Wuxi, Jiangsu China; 9 Jiangsu Yize Biotechnology Co., Ltd., Zhongke Innovation Center, Changzhou Science and Education Town, Changzhou, Jiangsu China; 10 Institutes for Translational Medicine, Soochow University, Suzhou, Jiangsu China; 11 Changzhou Medical Center, Nanjing Medical University, Changzhou, Jiangsu China

**Keywords:** Cancer, Tumour immunology

## Abstract

Immune checkpoint inhibitors (ICIs) are widely used in clinical oncology owing to their effectiveness against various tumors. However, by enhancing their immune responses, these inhibitors can trigger immune-related adverse events (irAEs) affecting various organ systems. Notably, pulmonary complications, particularly immune checkpoint inhibitor-related pneumonitis (ICIP), have emerged as one of the leading causes of treatment-related mortality in patients receiving ICIs. Given the limitations of current ICIP treatments, mesenchymal stem cells (MSCs) represent a promising therapeutic strategy owing to their immunomodulatory properties and ability to promote tissue repair. This article reviews recent advances in ICIP and proposes the potential applications of MSC therapy, emphasizing the need for further research into its efficacy and safety to improve ICIP management.

## Facts

- Immune checkpoint inhibitor-related pneumonitis (ICIP) is a severe immune-related adverse event that requires clinical attention.
- Current evidence suggests that immune cell imbalance, the production of autoantibodies, and cytokine storms may be key mechanisms behind ICIP.
- Glucocorticoids are the current first-line treatment, but evidence-based standards for treatment-resistant ICIP are still lacking.

## Open questions

- Do mesenchymal stem cells (MSCs) have significant immune-regulatory and tissue-repair potential? Can they serve as a next-generation treatment strategy for ICIP?
- In patients who have previously received PD-1/PD-L1 inhibitors, how does MSC intervention influence long-term outcomes such as lung function, tumor control, and the risk of secondary malignancies?

## Introduction

Immune checkpoint inhibitors (ICIs), particularly PD-1/PD-L1 inhibitors, have become a cornerstone of oncology treatment and are widely used for malignancies such as lung cancer, melanoma, renal cell carcinoma, and other tumors [1]. These inhibitors enhance the immune response by blocking the interaction between the PD-1 checkpoint and its ligand, PD-L1, allowing T cells to mount a strong attack on tumor cells. However, this immune activation can also disrupt normal immune tolerance, causing immune-related adverse events (irAEs) that affect multiple organ systems and can be life-threatening [2, 3]. Among irAEs, ICI-related pneumonitis (ICIP) is the most common pulmonary toxicity associated with ICI therapy and a major contributor to ICI-related mortality, accounting for 28% of deaths linked to these inhibitors [4, 5]. ICIP presents with a range of clinical symptoms, from dyspnea and cough to chest pain, and in severe cases, respiratory failure and death [1, 6, 7]. However, its pathogenesis remains elusive, and treatment options are limited. Mesenchymal stem cells (MSCs) have demonstrated significant therapeutic potential in various immune and inflammatory diseases owing to their strong ability to regulate immune and inflammatory responses [8]. In addition, MSCs have been reported to exhibit protective effects in immune-mediated diabetes and hepatitis [9, 10].

Here, we provide an in-depth summary of the recent advances related to ICIP, exploring the potential of MSC therapy in managing ICIP and aiming to offer valuable references and insights for future management of ICIP.

## ICIP

### Incidence and characteristics

Previous clinical trials have reported that the incidence of ICIP in patients with cancer treated with anti-PD-1/PD-L1 therapies is generally below 10%, mostly between 3% and 5% [5, 11–13]. The latest EDGE-Gastric phase II multicenter study showed that domvanalimab (an Fc-silent anti-TIGIT antibody) combined with zimberelimab (anti-PD-1)) had an incidence of ICIP of ~5%, with no events of grade ≥3 observed [14]. In real-world practice, the incidence of ICIP may be higher owing to a more diverse patient population and the presence of more potential risk factors, such as different types of cancer, different treatment regimens, and patients with different underlying diseases. Recent prospective and retrospective real-world studies reported incidences of ICIP of 16.7%, 30.5%, and 9.5% [15–17]. ICIP can develop from hours to 1–2 years after initiating anti-PD-1/PD-L1 therapy, though most cases occur within 6 months, with a median onset of ~2–3 months [11, 18]. Underlying lung diseases, or a history of lung disease, such as chronic obstructive pulmonary disease (COPD)/interstitial lung disease (ILD), are important risk factors for developing ICIP. For example, Atchley et al. showed that baseline fibrosis on chest CT is associated with ICIP (adjusted odds ratio, aOR 6.61), whereas obstructive lung diseases have an aOR of 2.79 [17]. Wang et al. reported that in lung cancer with COPD, ICIs are associated with a significantly higher ICIP rate (20.40% vs 11.48%) but improved PFS/OS. Mechanistically, COPD-related chronic inflammation and lung tissue fragility elevate pneumonitis risk, whereas a hotter TME (greater CD8 + T-cell and PD-L1+ macrophage infiltration) plus higher TMB and PD-1/PD-L1 expression enhances responsiveness to ICIs [19]. The incidence of ICIP is higher in patients treated with PD-1 inhibitors than in those treated with PD-L1 inhibitors [20, 21]. A meta-analysis of 29 studies involving 4639 patients with breast cancer treated with ICIs found PD-1 inhibitors to be linked to a higher ICIP incidence than PD-L1 inhibitors (5% vs 2%) [11]. This heightened risk may result from elevated PD-1 expression on various immune cells, leading to broader immune system activation and a greater probability of severe ICIP events. Notably, while both nivolumab and pembrolizumab are PD-1 inhibitors, research suggests pembrolizumab may carry a higher ICIP risk [17], potentially owing to differences in administration methods, molecular structures, or binding properties. ICIP incidence is significantly lower when using CTLA-4 inhibitors compared to PD-L1 inhibitors [1]. However, a recent multicenter real-world study on non-small cell lung cancer (NSCLC) reported that the incidence of ICIP in the CTLA-4 group was 18.5%, compared to 11.3% in the non-CTLA-4 group (pembrolizumab or atezolizumab plus platinum-based chemotherapy) (*P* = 0.058), a difference that did not reach statistical significance. The analysis revealed no significant differences in progression-free survival (PFS) or overall survival (OS) between patients with and without ICIP. Notably, while patients with mild to moderate (grades 1–2) ICIP had significantly longer PFS than those with severe (grade ≥3) ICIP (11.2 vs 2.6 months), OS showed a trend toward improvement [22]. Combination therapy can also increase this incidence. A meta-analysis of patients with various solid tumors treated with PD-1/PD-L1 inhibitors found that ICIP incidence was notably higher with combination immunotherapy (10%) compared to monotherapy (3%) [23]. When PD-1/PD-L1 inhibitors are combined with CTLA-4 inhibitors, the incidence of ICIP is also higher than that with monotherapy [1, 12]. Moreover, although smoking history, infection, and PD-L1 expression are considered risk factors for ICIP, higher baseline interleukin-8 (IL-8) levels are associated with a lower incidence [1, 4, 7].

Conversely, the incidence of ICIP is relatively lower in patients with non-lung tumors, such as biliary tract tumors, urothelial carcinoma, head and neck squamous cell carcinoma, and malignant melanoma [15, 24, 25]. The TOPAZ-1 study, evaluating patients with advanced or unresectable biliary tract cancer, reported an ICIP incidence of 0.9% with the durvalumab + glucocorticoids (GC) regimen, with 0.3% for grade 3 or higher, showing no notable difference from the chemotherapy-only regimen [26]. A prospective observational study enrolled 126 patients receiving ICI therapy; the incidence of ICI-related pulmonary toxicity was 16.7% (21/126). In the lung cancer subgroup, it was 25.0% (13/52) as compared with 10.8% (8/74) in non-lung cancer patients [15]. A recent study showed that patients with lung tumors are more than twice as likely to develop ICIP compared to those without lung tumors. This may be related to the localized inflammatory response and disruption of immune tolerance triggered by the tumor [3].

### Mechanism

**Fig. 1:**
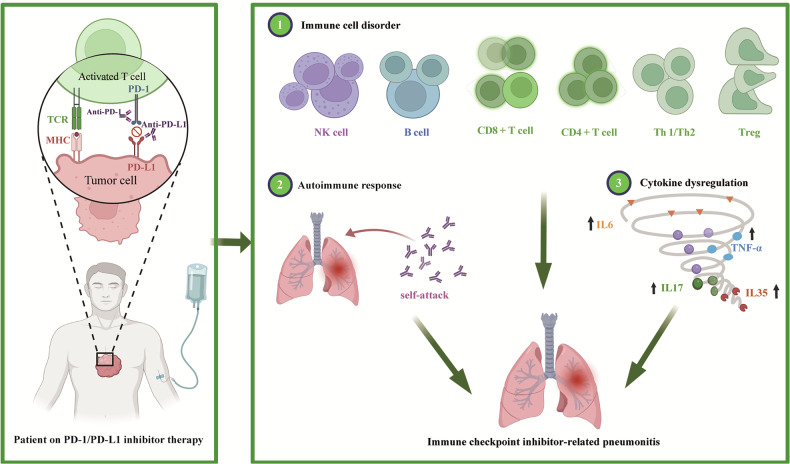
Illustration of the pathogenesis of ICIP. PD-1/PD-L1 inhibitors block the interaction between PD-1 and PD-L1, releasing the inhibition on T cells and activating the immune system to eliminate tumor cells. However, this immune activation disrupts immune self-tolerance, leading to immune cell imbalance, autoimmune reactions, and elevated levels of pro-inflammatory cytokines, which collectively contribute to ICIP development. ICIP immune checkpoint inhibitor-related pneumonitis.

Although the precise mechanism underlying ICIP is not fully understood, immune dysregulation triggered by PD-1/PD-L1 inhibitors likely plays a central role [27]. These inhibitors disrupt immune checkpoint pathways, thereby enhancing the attack of the immune system on tumor cells at the expense of self-tolerance. This heightened immune activity can destabilize the autoimmune microenvironment, leading to ICIP and a range of irAEs. The mechanisms of ICIP and immune microenvironment dysregulation involve several key processes (Fig. 1).

### Immune cell disorder

The imbalance of immune cell subsets is a significant feature of the immune microenvironment in patients with ICIP [28, 29]. In the inflammatory immune microenvironment of patients with ICIP, pro-inflammatory cells, such as CD8 + T cells, M1 macrophages, and activated effector CD4 + T cells, dominate. These cells actively drive lung inflammation and tissue damage by secreting large quantities of pro-inflammatory factors (such as IFN-γ and TNF-α) [28]. Previous studies have observed a significant increase in CD4+ and CD8 + T lymphocytes in the lung tissue and bronchoalveolar lavage fluid (BALF) of patients with ICIP and animal models [29–31]. Upon antigen recognition and cytokine signaling in secondary lymphoid organs, naive CD4 + T cells differentiate into specialized helper T-cell subsets, such as Th1, Th2, and Th17. In the context of ICIP, dysregulation of these subsets (such as increased Th1 and Th17 cell numbers) contributes to disease progression. In contrast, the proportion of anti-inflammatory cells, including regulatory T cells (Tregs) and M2 macrophages, is significantly reduced, potentially exacerbating the overactivation of effector T cells and impairing their roles in immune regulation and tissue repair [28, 29, 32].

Further, in severe ICIP, the number and activity of neutrophils are elevated in the bronchoalveolar lavage fluid (BALF) [33]. Zhang et al. observed that patients with ICIP had significantly higher dendritic cell type 2 (DC2) cell number, which exhibited enhanced signaling to immune cells, potentially promoting the presentation of autoantigens [34]. B cells and natural killer (NK) cells, as integral parts of the immune system, are also implicated in ICIP development following PD-1/PD-L1 inhibitor administration [29]. PD-1/PD-L1 blockade therapy may activate NK cells and induce inflammatory responses, subsequently leading to lung tissue damage.

### Autoantibody-mediated immune response

Recent research suggests that irAE onset might be associated with increased autoantibody levels and the immune responses they trigger. Autoantibodies such as antinuclear and rheumatoid factor antibodies may either pre-exist before ICI therapy or be generated after immunotherapy [27, 32]. Osorio et al. observed that thyroid dysfunction in patients receiving anti-PD-1 therapy may be related to the presence of antithyroid antibodies [35]. In addition, antibodies such as anti-GNAL and anti-ITM2B are linked to hypophysitis induced by ICI therapy [36]. Although the specific autoantibodies related to ICIP development are still under investigation, preliminary studies have offered some indications. For instance, patients with NSCLC treated with a PD-1 inhibitor have shown a significant increase in IgM antibodies against the cholinergic receptor nicotinic gamma subunit (ACHRG) preceding ICIP development. Moreover, those who developed ICIP had markedly higher anti-ACHRG antibody levels than those who did not. This finding implies that pre-existing autoantibodies against ACHRG might play a role in pneumonitis development [37, 38]. Further studies have revealed that in patients with ICIP undergoing anti-PD-1 therapy, anti-CD74 antibody levels notably increase post-treatment. In contrast, no significant changes in anti-CD74 antibody levels were detected in the control group of patients who remained pneumonitis-free [36]. These findings underscore the potential role of autoantibodies in ICIP pathogenesis and the need for further research to elucidate their mechanisms and clinical implications.

### Dysregulation of inflammation-related cytokines

The dysregulation of inflammation-related cytokines is another important cause of ICIP. IL-6, a cytokine involved in immune response, inflammation regulation, and the activation and differentiation of T and B cells, has been linked to certain irAEs [29, 39]. In patients with ICIP, IL-6 levels are significantly elevated and closely related to the severity and prognosis, marking it as a key inflammatory factor [29, 40]. Suresh et al. observed that in the BAL fluid of patients with ICIP, despite a higher number of cells expressing IL-1β detected using flow cytometry, the soluble IL-1β levels in the BAL fluid were lower, suggesting that inflammation intensity may influence the complex dynamic in IL-1β levels [41]. Furthermore, an increase in C-reactive protein (CRP), IL-17, and IL-35 levels has been associated with ICIP, indicating a broader cytokine dysregulation in the immune environment of patients with ICIP [40, 42]. Table 1 summarizes the major changes in cytokines in ICIP [30, 40–44]. Various inflammatory factor inhibitors, such as infliximab (TNF-α inhibitor) and tocilizumab (IL-6 inhibitor), have effectively treated ICIP [45]. These findings underscore the significant immune dysregulation in the pathophysiological mechanisms of ICIP and suggest that monitoring these cytokines could provide valuable insights into disease progression and treatment response.

**Table 1:** Major changes in cytokines in ICIP.

Patient details	Testing	Changes in major cytokine(s)	Ref.
28 ICIP patients	Serum	↑IL-6, IL-8, IL-10,TNF-α	[30]
87 ICIP and 87 lung cancer patients	Peripheral blood	↑IL-6, IL-10	[40]
12 ICIP patients and 6 NSCLC patients	BALF	↑IL-12p40, CXCL-10, CCL17; ↓ IL-8	[41]
13 ICIP patients and 20 NSCLC patients	Blood specimens	↑IL-17 A, IL-35 (decrease after recovery)	[42]
12 ICIP patients, 12 healthy individuals, 12 lung cancer patients, 12 patients with other ILD	BALF and serum	↑IL-6, IL-17 A, IFN-γ (in BALF)	[43]
56 ICIP patients	Peripheral blood	↑IL-6, IL-10, hsCRP	[44]

*BALF* bronchoalveolar lavage fluid, *ICIP* immune checkpoint inhibitor-related pneumonitis, *ILD* interstitial lung disease, *NSCLC* non-small-cell lung cancer, *hsCRP* high-sensitivity C-reactive protein, *IFN-γ* interferon-gamma.

### Bronchoalveolar lavage (BAL) findings of ICIP

The BAL examination aids in early ICIP identification, thereby providing better treatment and management strategies for patients. According to guidelines, when a patient reaches ICIP Grade 2, performing bronchoscopy in conjunction with BALF analysis is recommended to comprehensively assess lung conditions and guide subsequent treatment [7, 46]. The BALF in ICIP consistently exhibits a T cell-centered inflammatory phenotype: lymphocytosis is commonly observed [30, 47]; significant activation of CD8 effector memory and tissue-resident memory T cells (Tem/Trm), as well as CD4 T follicular helper–like (Tfh-like), Th1, and Th17-related signals. Conventional dendritic cells (cDC1/cDC2) show enhanced MHC-II antigen presentation; alveolar macrophages relatively decrease. While inflammatory monocyte-derived/intermediate macrophages increase [34, 41], at the chemokine/cytokine level, the CXCL13/CXCR4 axis, TNF-α, IFN-γ, and TWEAK are markedly upregulated [34, 41]. Moreover, in clinical differentiation, elevated IL-6 in BALF aids in distinguishing ICIP from tumor progression or non-immune interstitial lung disease (ILD) [48]. Moreover, multiple studies suggest that IL-6 may serve as a potential predictive clinical indicator and management strategy for patients with ICIP [7, 30]. However, as elevated IL-6 levels have also been found in the BALF of infection patients, the IL-6 levels in BALF may not be the optimal indicator to differentiate ICIP from infection [49].

### CT patterns of ICIP

The imaging characteristics are of significant importance for the diagnosis and treatment of ICIP. The imaging characteristics of ICIP mainly include ground-glass opacity (GGO), consolidation, interlobular septal thickening, and reticular opacity. Among these, while GGO is the most common imaging manifestation, with studies indicating that GGO appears in 96.1% of cases, consolidation is observed in 53.9% of patients [50]. The most common imaging pattern of ICIP is organizing pneumonia (OP), characterized by the presence of multiple patchy consolidations and GGOs in the peripheral regions of the lungs. In addition, the reverse halo sign may be observed in imaging. Other common imaging patterns include nonspecific interstitial pneumonia (NSIP), hypersensitivity pneumonitis (HP), acute interstitial pneumonia (AIP), and diffuse alveolar damage (DAD) [51–53]. The clinical symptoms and lung damage associated with the AIP pattern are relatively severe and are typically observed in patients with severe ICIP [53, 54]. These imaging features are closely associated with prognosis; for instance, ICIP patients with lesions around tumors and presenting reticular opacities generally have a poorer prognosis, and Grade 1 and imaging improvement/resolution are associated with better survival [50, 51]. The imaging characteristics also aid in distinguishing ICIP from radiation pneumonitis (RP). RP is typically confined to high-dose radiation areas, with sharp boundaries and geometric shapes, whereas ICIP presents as bilateral multifocal patterns that are non-dose-dependent and have unclear margins [50, 51]. In addition, when chest imaging reveals changes such as mediastinal or hilar lymphadenopathy and heterogeneous pulmonary density, consideration should be given to differentiating ICIP from immune-related pulmonary nodules (another form of pulmonary toxicity associated with ICIs).

In recent years, advancements in radiomics are also expected to enhance the diagnosis, risk prediction, and prognosis evaluation of ICIP. Research by Chen et al. showed that radiomic features can predict the risk of ICIP to some degree [55]. Scientists integrated radiomics and deep learning to create a predictive model using clinical data and pretreatment CT images to assess ICIP risk in lung cancer patients undergoing immunotherapy [56]. Their study analyzed baseline CT images and clinical data from 24 patients who developed ICIP and 24 controls who did not, employing multimodal data, two-stage transfer learning, and contrastive learning. The model achieved an area under the receiver operating characteristic curve (AUC) of 0.918 and an accuracy of 0.920. Tingyue Luo and colleagues developed a CT imaging-based radiomics machine learning model to differentiate between ICIP and other types of pneumonia. The study included 238 patients, and the model demonstrated high accuracy. This model provides rapid, non-invasive etiological support for pneumonia in clinical settings [57]. These results highlight the potential of CT radiomics in disease diagnosis. Further studies are required to confirm the clinical utility of these technologies in ICIP management.

### Diagnosis and grading of ICIP

ICIP presents with symptoms such as dyspnea, cough, chest pain, and fever, though some cases are identified solely by radiographic changes [7]. ICIP diagnosis relies on evaluating the patient’s history of ICI use, radiographic findings, and excluding disease progression, infections, or cardiac conditions. Clinicians should maintain a high index of suspicion for ICIP in patients on anti-PD-1/PD-L1 therapies who develop new pulmonary opacities or symptoms such as dyspnea, chest pain, cough, and fever. For patients with suspected pneumonitis, evaluation should be conducted using high-resolution chest CT and pulmonary function tests [46, 58]. Upon diagnosis, ICIP severity should be graded, and appropriate treatment initiated. The ICIP grading should be comprehensively evaluated based on the patient’s clinical symptoms, imaging findings, and rate of progression. Commonly used ICIP grading standards include the Common Terminology Criteria for Adverse Events (CTCAE) issued by the National Institutes of Health and the National Cancer Institute, as well as the grading standards from the management guidelines for irAEs published by the National Comprehensive Cancer Network (NCCN), the American Society of Clinical Oncology (ASCO), and the European Society for Medical Oncology (ESMO) [6, 46, 59, 60]. These guidelines standardize ICIP diagnosis and management, ensuring timely and effective patient care. Table 2 summarizes the commonly used ICIP grading standards.

**Table 2:** Grade standard for ICIP.

Grade	Guidelines standard	CTCAE v6.0 standard
G1	Asymptomatic; confined to <25% of lung parenchyma	Symptomatic; clinical or diagnostic observations only; intervention not indicated
G2	Presence of new/worsening symptoms; involving 25–50% of lung parenchyma; medical intervention indicated;	Symptomatic; medical intervention indicated; limiting instrumental ADL
G3	Severe symptoms; involves all lung lobes or >50% of lung parenchyma; requires hospitalization or oxygen; limiting self-care ADL; oxygen indicated	Severe symptoms; oxygen indicated; limiting self-care ADL
G4	Life-threatening respiratory compromise; ARDS	Life-threatening respiratory compromise; urgent intervention indicated
G5		Death

*ADL* activity of daily living, *ARDS* acute respiratory distress syndrome.

### ICIP treatment

ICIP treatment should be customized according to the severity grade, with corticosteroids serving as the cornerstone of therapy. For ICIP patients, guidelines recommend graded management, suggesting dynamic evaluations based on changes in conditions to timely adjust the grading and corresponding treatment measures [6, 58, 61]. For Grade 1 ICIP, patients’ conditions should be closely monitored, and ICIs should either be paused or continued under monitoring. For Grade 2 ICIP, ICIs should be discontinued, and GC treatment should be initiated. The ESMO recommends prednisone at a dose of 1 mg/kg/day, whereas the NCCN, ASCO, and SITC suggest a dosage of 1–2 mg/kg/day. Furthermore, using pulsed corticosteroid therapy has been reported [16, 62].

NCCN, ASCO, and ESMO recommend treating with empirical antibiotics when infection cannot be ruled out. If symptoms do not improve within 48–72 h of steroid therapy, escalation to Grade 3–4 treatment protocols is required. ICIs may be resumed after treatment if clinical and radiographic responses return to Grade 1 or better. In patients with grade 2 ICIP, ICI rechallenge is associated with a significant OS benefit compared with no rechallenge. Nevertheless, the risk of recurrent ICIP is substantial—~40% across studies—warranting a careful risk–benefit assessment and close monitoring [63, 64]. Recent retrospective analyses show that carefully monitored ICI rechallenge/continuation in grade 1–2 ICIP confers significant PFS and OS benefits relative to permanent discontinuation, whereas ICIP recurs in roughly 50% of rechallenged cases without a significant impact on PFS or OS [65]. However, the reuse of ICIs is not advised in patients with poor glucocorticoid responsiveness and significant post-treatment declines in pulmonary function [66].

For Grades 3–4 ICIP, guidelines recommend permanently discontinuing ICIs.

If no symptom improvement is observed within 48 h after treatment with glucocorticoids (GCs), immunosuppressive therapy may be added, including tocilizumab, infliximab, intravenous immunoglobulin (IVIG), mycophenolate mofetil, or cyclophosphamide. In the latest updated 2025 NCCN guidelines, IVIG and mycophenolate mofetil are listed as preferred options. Other recommendations include tocilizumab and infliximab [46]. Tocilizumab, a monoclonal antibody targeting the interleukin-6 (IL-6) receptor, has been reported to be effective in the treatment of irAEs [67, 68]. A case has been reported in which a patient with NSCLC developed symptoms of immune pneumonitis after receiving atezolizumab. The patient was treated with tocilizumab, resulting in significant symptomatic relief within 2 days and a significant decrease in CRP levels [69]. A retrospective study showed that most of the 34 patients with irAEs, including ICIP, experienced relief after tocilizumab treatment [70]. However, more experiments are still needed to verify its effectiveness and safety. The latest NCCN guidelines also state that owing to the increased risk of gastrointestinal perforation associated with toclizumab, assessing patients for a history of clinically active diverticular disease prior to initiating therapy and using caution in these patients is important [46].

TNF-α inhibitor infliximab is effective in treating ICI-related hepatitis [71]. A case report describes a life-threatening patient with ICIP who received intravenous methylprednisolone along with infliximab. In the following days, the patient’s clinical condition improved significantly [72]. It should be noted that evidence for the efficacy of infliximab in ICIP is inconsistent, and its use should be approached cautiously [46]. A patient developed ICIP after receiving pembrolizumab treatment, did not respond to corticosteroid therapy, and eventually needed mechanical ventilation. Subsequently, the patient was treated with infliximab, resulting in rapid improvement in respiratory status, with chest CT scan showing a reduction in ground-glass opacities. However, the patient’s pneumonitis worsened again shortly after [73]. In addition, IL-17, IL-12, and IL-23 inhibitors are effective in treating immune-related skin toxicities, colitis, and other immune-related adverse events, suggesting that targeting these inflammation-related factors may be an effective option for treating ICIP [45].

For corticosteroid-sensitive ICIP, corticosteroids should be tapered gradually after treatment becomes effective. For Grade 2 ICIP, the total duration of corticosteroid therapy is recommended to be 4–6 weeks. For Grade 3–4 ICIP, the total duration of corticosteroid therapy is recommended to be at least 6 weeks [46, 59]. As for Grade 2 ICIP, the latest NCCN guidelines recommend considering mycophenolate mofetil as a steroid-sparing immunosuppressant for recurrent pneumonitis during steroid tapering owing to its clinical benefits in avoiding steroid dependence [46].

### Potential of MSC therapy for ICIP

Nowadays, determining how to select alternative treatments for corticosteroids, refractory or corticosteroid-dependent ICIP, and exploring new therapeutic approaches, especially the use of biological agents, remains an unresolved issue in ICIP management. MSC therapy has emerged as a promising approach in regenerative medicine, offering potential solutions for repairing damaged tissues and treating various diseases. MSCs can be obtained from various tissues, including bone marrow, adipose tissue, and umbilical cord [74]. Their potent immunomodulatory functions and paracrine mechanisms contribute significantly to their therapeutic effects [8, 75, 76]. Moreover, commonly used adult stem cells have advantages such as easy isolation, rapid proliferation, genetic stability, and no ethical concerns, significantly promoting their clinical applications. We, therefore, integrate evidence from related ICI toxicities, non-ICI lung injuries, and immune-inflammatory diseases, alongside putative mechanisms, to explore the theoretical feasibility and rationale for MSC therapy in ICIP (Table 3).

**Table 3:** Existing evidence of MSC therapy for ICIP.

Type	Strength	Source of evidence	Description
ICIP-specific			Currently lacking
MSCs in irAEs	Partial	Preclinical research	Diabetes and hepatitis induced by ICIs
MSCs in non-ICI lung injuries	Morerate	Clinical trials; preclinical research	Various acute and chronic lung diseases: acute Lung Injury/ARDS; bacterial pneumonia/viral pneumonia; asthma; IPF
MSCs in immune-inflammatory diseases	Morerate	Clinical trials; Preclinical research	Various inflammatory immune diseases, including renal injury, Crohn’s disease, GVHD, osteoarthritis, RA, and IBD
Mechanistic evidence	Strong	Clinical trials; Preclinical research	Regulation of immune cell/immune factor balance; damage repair and fibrosis prevention

*MSCs* mesenchymal stem cells, *ARDS* acute respiratory distress syndrome, *IPF* idiopathic pulmonary fibrosis, *GVHD* graft-versus-host disease, *IBD* inflammatory bowel disease, *RA* rheumatoid arthritis.

### Evidence for MSCs in irAEs

MSCs have demonstrated protective effects in certain irAEs. Kawada-Horitani et al. constructed a mouse model of type 1 diabetes induced by ICI. They found that the injection of MSCs significantly reduced the incidence of type 1 diabetes and played an important role in protecting islet β cells [10]. The experimental results showed that the repeated use of ICI led to immune cells infiltrating islet β cells, reducing the β cell area and insulin content in the pancreas. However, after the injection of MSCs, this infiltration by immune cells was inhibited. Moreover, the researchers used PKH dye to label MSCs. They found that the MSCs primarily accumulated in lung tissue, while their protective effects on the pancreas likely relied more on the secretion of cytokines or exosomes. Furthermore, a research team combined the anti-inflammatory properties of MSCs with the targeting advantages of nanotechnology to develop an immunoregulatory nanoparticle (MSC-PD-L1+NPs) [9]. The team constructed this system by encapsulating poly (lactic-co-glycolic acid) (PLGA) nanoparticles with the plasma membrane of MSCs overexpressing PD-L1. It was designed to treat irAEs induced by ICIs. In a mouse model, MSC-PD-L1+ NPs precisely targeted inflammatory sites in the liver. By interacting with activated T cells and macrophages, they significantly suppressed excessive activation, reduced the secretion of inflammatory cytokines (such as IL-6), effectively alleviated liver injury, and improved key liver function indicators. More importantly, these nanoparticles predominantly accumulated in liver tissues without significantly affecting the antitumor efficacy of ICIs, thereby avoiding the potential risks of traditional immunosuppressants that may compromise antitumor immunity. These findings suggest that MSCs may help protect against irAEs with potentially limited tumor-promoting risks, providing a rationale for ICIP investigation.

### Evidence for MSCs in non-ICI lung injuries

MSCs have additional advantages in the treatment of pulmonary diseases. After intravenous injection, many MSCs accumulate in the lungs due to the filtering effect of blood vessels, which is referred to as the “first-pass effect.” Studies have observed that this phenomenon is mainly attributed to the mechanical obstruction caused by the relatively large size of the cells, rather than migrating through rolling and adhesion-like white blood cells. Although the first-pass effect limits the distribution of MSCs to other tissues throughout the body to some extent, it provides a direct and convenient approach for treating lung injuries [77, 78]. MSC therapy demonstrates promising therapeutic potential for pneumonia-induced lung injury (Table 4) [79–87]. Therefore, the use of MSCs for the treatment of ICIP is considered a feasible option.

**Table 4:** Research on pneumonia-induced lung injury treated with MSC-based therapy.

Details	Lung injury type	Cell type	Model	Outcomes	Ref.
Preclinical research	Antibiotic-resistant Klebsiella pneumoniae	BM, AD, UC-MSCs	Rat	Promote recovery; naive MSCs show better outcomes than pre-activated	[79]
Preclinical research	Radiation pneumonia	BM-MSCs	Mice	Decrease ROS and inflammation; delay fibrosis	[80]
Preclinical research	Radiation pneumonia	BM-MSCs	Mice	Decrease collagen deposition; regulate inflammatory response	[81]
Preclinical research	Radiation pneumonia	AD-MSCs	Rat	Decrease pulmonary fibrosis; anti-inflammatory effects	[82]
Phase 1/2a randomized controlled clinical trial	Viral pneumonia	UC-MSCs	Human	Safe infusion; increase survival and decrease cytokines	[83]
Phase 2a randomized controlled clinical trial	Viral pneumonia	UC-MSCs	Human	Safe; effective for severe lung injury	[84]
Phase 2 double-blind controlled trial	Viral pneumonia	UC-MSCs	Human	Decrease mortality vs standard care; decrease inflammation	[85]
Phase 1 clinical trial	Viral pneumonia	UC-MSCs-derived exosomes	Human	Nebulization of MSC-derived exosomes is safe and effective	[86]
Phase 1 randomized controlled clinical trial	Viral pneumonia	hPMSC-sEVs	Human	hPMSC-sEVs reduce mortality and extend survival time	[87]

*AD* adipose tissue, *BM* bone marrow, *DB* double-blind, *IL-10* interleukin-10, *MSC* mesenchymal stem cell, *RCT* randomized controlled trial, *ROS* reactive oxygen species, *TNF-α* tumor necrosis factor-alpha, *UC* umbilical cord, *hPMSC-sEVs* placental mesenchymal stem cell-derived small extracellular vesicles.

Ref [81]: Low-dose MSCs can reduce collagen deposition, regulate the inflammatory response, and prove effective in treating radiation-induced lung injury.

Ref [82]: MSCs significantly reduce the severity of pulmonary fibrosis and exhibit good anti-inflammatory effects.

Ref [83]: The MSCs infusion was safe, reduced inflammatory cytokine levels, significantly increased patient survival rates compared to the control group, and shortened recovery time.

Ref [84]: MSCs were safe in the treatment of lung damage in severely ill COVID-19 cases and effectively improved lung lesions.

Ref [85]: Critically ill patients receiving MSCs treatment had a significantly lower mortality rate compared to those receiving standard treatment, and inflammatory markers were notably reduced in the MSCs group.

### Evidence from other immune-mediated inflammatory diseases

Numerous clinical and preclinical studies have demonstrated the efficacy of MSC therapy in various inflammatory and immune diseases, such as inflammatory bowel disease and graft-versus-host disease [8, 88, 89]. Despite distinct immunologic triggers, these conditions (e.g., Crohn’s disease and GVHD) share key features with ICIP: all are T cell-mediated immunoinflammatory states characterized by upregulated proinflammatory cytokines, and initial management commonly relies on corticosteroid induction. MSCs exert their effects primarily through multi-targeted immunomodulatory mechanisms, achieving a comprehensive outcome of suppressing inflammation, promoting immune homeostasis, and facilitating tissue repair, which provides a reference for ICIP.

### Potential mechanisms of MSCs in ICIP treatment

**Fig. 2:**
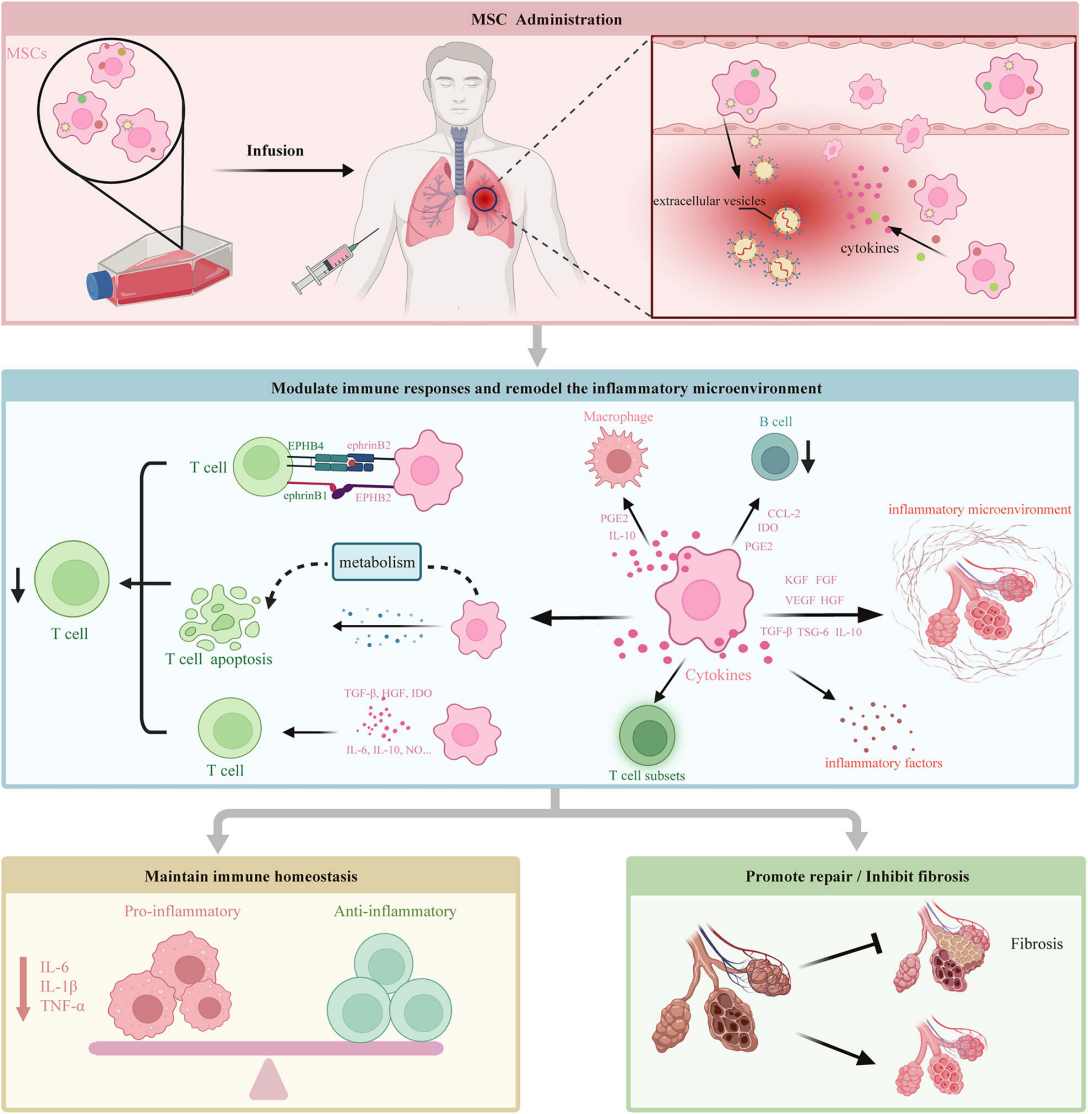
Mechanisms of MSCs in ICIP treatment. After intravenous injection, MSCs regulate T-cell metabolism and apoptosis through cell contact, the release of extracellular vesicles, and various immunomodulatory factors, reducing aberrant T-cell activation and excessive immune responses, thereby alleviating tissue injury. Moreover, MSCs modulate various immune cells and inflammatory factors, lowering pro-inflammatory cytokines such as IL-6, IL-1β, and TNF-α, enhancing anti-inflammatory responses, and helping to maintain immune homeostasis. Finally, by secreting growth factors and inhibiting fibrotic pathways, MSCs promote lung tissue repair and prevent fibrosis, providing a novel therapeutic strategy for ICIP. ICIP immune checkpoint inhibitor-related pneumonitis, MSCs mesenchymal stem cells.

The dysregulation of the immune system caused by ICIs is a key mechanism underlying ICIP development. MSCs possess powerful regulatory abilities that contribute to the maintenance of immune homeostasis (Fig. 2).

#### Regulation of immune cell balance

Under the stimulation of pro-inflammatory factors, MSCs exhibit a significant ability to suppress T-cell proliferation through various mechanisms, including cell-cell contact, secretion of immunosuppressive factors, and metabolic pathways. MSCs express EPHB2 and ephrinB2 on their surface, which interact with ephrinB1 and EPHB4 on T cells, thereby inhibiting T-cell function and enhancing IDO expression in MSCs [90]. MSCs inhibit T-cell activation and proliferation through PD-L1 and FasL while modulating cell cycle-related proteins via HLA-G1, TGF-β, and HGF to arrest the T-cell cycle in the G1 phase, thereby exerting immunosuppressive effects [90, 91]. Furthermore, in an inflammatory environment, MSCs recruit T cells to their proximity by secreting chemokines and suppress T-cell proliferation and activation through the expression of IDO [8]. In addition, MSCs induce T-cell apoptosis and inhibit their activity by regulating tryptophan metabolism, the Fas/FasL pathway, and galectin secretion [90]. MSCs directly regulate T cells and exert immunosuppressive effects by promoting the expansion and functional enhancement of TRegs [91]. MSCs can also regulate B-cell and T-cell subsets to exert immunomodulatory effects, and their potential mechanisms for treating ICIP are shown in Fig. 2 and Table 5 [76, 90].

**Table 5:** The regulatory role of MSCs in immune cells.

Immune cells	Changes	Promoting effect	Inhibiting effect
T cell	↑[49]	Proliferation/activation	Apoptosis
Treg	↓[29]	Proliferation/activation/differentiation/regulatory functional	
Th1	↑[29]	Anti-inflammatory factors	Proliferation/activation/pro-inflammatory factors
		Apoptosis	
Th17	↑[32]	Anti-inflammatory factors/Treg conversion	Differentiation/activation
NK	↓[28]		Proliferation/activation
B	↑[28]	Non-activated B cells	Differentiation/proliferation/activation/Maturation/Antibody production
Macrophage	↑/↓[28, 33, 34]	M2 polarization	Differentiation/activation/M1 polarization
Netrophil	↑[33]		Recruitment/migration/activation extracellular trap formation/protease secretion
DC	↑*[34]	Transform into an anti-inflammatory phenotype	Differentiation/proliferation/maturation/Activation/Migration

*Compared to patients with COVID-19, the proportion of DC2 cells in the bronchoalveolar lavage fluid of ICP patients is higher.

#### Regulation of immune factor balance

MSCs can regulate the balance of inflammatory factors, thereby maintaining normal immune system function and potentially playing a key role in the treatment of ICIP. During ICIP development, inflammatory factors are dysregulated. As previously mentioned, IL-6 is involved in immune responses and inflammation regulation, and it is a key inflammatory factor in the pathogenesis of ICIP [29, 39, 40, 69]. In the context of inflammation, MSCs can significantly reduce the levels of various inflammatory factors, including IL-6 [39, 82]. Furthermore, MSCs have broad regulatory effects on inflammatory factors. In the process of lung injury, MSCs can reduce the levels of various pro-inflammatory mediators, such as IL-1β, TNF-α, and IFN-γ, while increasing the levels of anti-inflammatory cytokines, including IL-10, basic fibroblast growth factor (bFGF), and TGF-β, thereby effectively preventing excessive inflammatory responses [92]. Tumor necrosis factor-stimulated gene 6 (TSG-6) is an important anti-inflammatory factor that plays a significant role in tissue-protective properties mediated by MSCs. Under the stimulation of inflammatory signals, MSCs secrete TSG-6 to modulate the immune microenvironment, suppress inflammation, and promote tissue repair [93, 94]. MSCs or their extracellular vesicles (containing TSG-6) can alleviate pulmonary inflammation and improve lung injury [95, 96]. Furthermore, MSC-derived TSG-6 plays a positive role in various inflammatory diseases [97–99]. Therefore, administering MSCs that enhance TSG-6 secretion offers a promising therapeutic strategy for ICIP.

#### Damage repair and fibrosis prevention

MSCs exert immune-regulatory effects, repair tissue damage, and inhibit the occurrence of fibrosis, potentially providing important support for lung regeneration in patients with ICIP. They promote tissue repair by releasing various bioactive molecules, including keratinocyte growth factor (KGF), which aids in the repair of alveolar epithelial cells; vascular endothelial growth factor (VEGF), which enhances vascular stability and promotes neovascularization; hepatocyte growth factor (HGF); and fibroblast growth factor (FGF) [92, 100]. Moreover, MSCs can regulate key fibrotic signals in the damaged microenvironment through paracrine and exosomal mechanisms, inhibit the proliferation of fibroblasts, and modulate the composition and structure of the extracellular matrix, thereby reducing the occurrence of pulmonary fibrosis [92, 100, 101].

MSCs exhibit remarkable plasticity. Rather than being passive participants in the injured microenvironment, they actively sense changes in their surroundings and adapt their secretory profiles accordingly [100, 102]. For instance, studies on cerebral ischemic injury models have shown that injecting MSCs at different time points post-injury yields significantly different effects: injections at 3 days can promote new cell generation, whereas injections at 10 days facilitate axonal remodeling [103]. The characteristics of MSCs may contribute to their role at different stages of ICIP, such as regulating immunity and controlling inflammation in the early phase, while promoting tissue repair and anti-fibrotic processes in later phases.

#### Exosome delivery

MSC-derived exosomes (MSC-Exos) represent a breakthrough in regenerative medicine, offering a cell-free alternative for stem cell therapy. MSC-Exos are 30–150-nm-thick extracellular vesicles enriched with bioactive components, such as proteins, lipids, DNA, and mRNA. They can be precisely delivered to target cells or tissues, exerting regulatory functions [104, 105]. With MSC-Exo advantages including low immunogenicity and efficient penetration of biological barriers, significant progress has been made in the fields of ischemic stroke, articular cartilage regeneration, neural function reconstruction, and cardiac repair [106]. A recent study revealed that human umbilical cord-derived MSC-Exos (UC-MSC-Exos) have a cardioprotective effect after acute myocardial infarction by reducing cell apoptosis and oxidative stress, significantly improving ventricular function [107]. In addition to traditional methods, MSC-Exos can be delivered directly to the lungs via nebulization to exert their immune modulation and tissue repair effects [108, 109]. This approach offers advantages, including high target specificity, non-invasive administration, and minimal side effects. A Recent study has investigated the safety and efficacy of nebulized human UC-MSC-Exos in the treatment of pulmonary fibrosis. Through mouse models and subsequent randomized double-blind clinical trials, the study showed that this therapy significantly improved survival rates in mice and enhanced lung function. In a cohort of 24 patients, lung function and quality of life also notably improved, with good tolerability and no severe adverse events reported. Additionally, exosomes exerted their effects by modulating immune responses and upregulating anti-fibrotic-related miRNAs, thereby alleviating pulmonary fibrosis [109].

Furthermore, exosomes can be engineered to precisely deliver specific cargo to targeted regions of the lungs, resulting in enhanced therapeutic efficacy and improved safety [105]. Zhang et al. explored the therapeutic effects of engineered exosomes (miR-486-RBD-MSC-Exo) for treating radiation-induced lung injury (RILI) and fibrosis. They significantly enhanced lung epithelial cell growth and migration while suppressing radiation-induced ferroptosis, leading to improved lung injury and fibrosis outcomes in mouse models and increased survival rates. Mechanistic studies revealed that exosomes alleviate fibrosis by inhibiting SMAD2 and activating the Akt pathway. This study underscores the potential of engineered exosomes in lung repair and offers new insights for future cell therapies [110].

### Safety of MSC therapy for ICIP

MSC treatment for ICIP requires attention to the tumor risks, as the relationship between MSCs and tumors is complex and controversial. MSCs may promote tumor progression by secreting cytokines and enhancing angiogenesis [111]. Furthermore, MSCs can be recruited to tumor regions under various cytokines, forming tumor-associated MSCs in the tumor microenvironment, which subsequently differentiate into cancer-associated fibroblasts (CAFs) [112, 113]. CAFs can secrete various pro-tumor factors (such as VEGF) and remodel the extracellular matrix, thereby supporting tumor growth and metastasis [112, 114]. The immunomodulatory role of MSCs is heavily influenced by microenvironmental factors. However, some cytokines (such as TNF-α and IFN-γ) are reported to exert dual roles on MSCs, and their concentrations may be an important influencing factor. For instance, appropriate stimulation with IFN-γ and TNF-α (such as 15 ng/ml TNF-α and 10 ng/ml IFN-γ) has been widely reported to enhance the immunoregulatory capacity of MSCs [115–117]. In contrast, high concentrations of IFN-γ and TNF-α inhibit the proliferation and differentiation of MSCs and lead to the upregulation of oncogenes (such as c-Fos and c-Myc) mediated by NFκB, thereby increasing the tumorigenic risk associated with MSCs [116, 118, 119]. Domenis et al. treated MSCs with 10–40 ng/ml of TNF-α and IFN-γ, and an increase in cytokine concentration led to significant alterations in MSC morphology and a marked decrease in proliferative capacity [120]. Furthermore, the role of temporal factors and the in vivo microenvironment in this process should not be overlooked [116]. In exploring MSC therapy for ICIP, closely monitoring these factors is essential to clarify their overall impact on the therapeutic potential of MSCs.

MSC therapy may also carry a risk of thrombosis. The mechanical retention of MSCs in the pulmonary microvasculature may raise concerns about vascular obstruction. However, no significant embolism has been observed following MSC injection [78]. MSCs, particularly AD-MSCs and UC-MSCs, may express high levels of tissue factor, triggering the coagulation cascade and potentially increasing the risk of thrombosis [119, 121]. Nevertheless, because of a lack of methodological standardization and inter-laboratory comparability, current evidence is insufficient to support the use of tissue factor as a stable, reproducible biomarker for predicting thrombotic risk [122, 123]. Most clinical trials to date have not reported significant risks of thrombosis [124, 125].

The potential interaction between MSCs and GCs is concerning. GCs may suppress the immunomodulatory capacity of MSCs, thereby weakening their therapeutic efficacy [126, 127]. Although endogenous GCs, under certain conditions, positively contribute to the mobilization and migration of MSCs [128]. Another study showed that under inflammatory conditions, where IFN-γ and TNF-α coexist, dexamethasone synergistically enhanced TSG-6 expression in MSCs [129]. In addition, in an acute lung injury model, MSCs showed enhanced anti-inflammatory effects in vitro [129]. Therefore, in clinical practice, carefully balancing glucocorticoid dosage with the effectiveness of stem cell therapy is essential for achieving optimal therapeutic outcomes.

The clinical application of MSCs has been widely carried out globally. Long-term observations and experiments have demonstrated that standardized intravenous infusion of MSCs is a highly safe therapeutic approach [124, 125, 130]. The most recent 2025 meta-analysis (36 RCTs) shows that intravenous MSCs are generally safe, infection events have slightly increased, and notably, a trend toward increased neurological events emerged. The authors speculate that this may be associated with the inflammatory–immune axis following the lung first-pass effect. This is worth careful attention when exploring MSC therapy for ICIP [131].

MSC-based therapies hold great promise for treating ICIP, as ongoing research and clinical advancements continue to explore their potential. Clinical studies are needed to observe and evaluate their safety, particularly in populations with advanced or uncontrolled tumors, where the balance between therapeutic efficacy and potential risks requires careful consideration and monitoring.

## Discussion

**Fig. 3:**
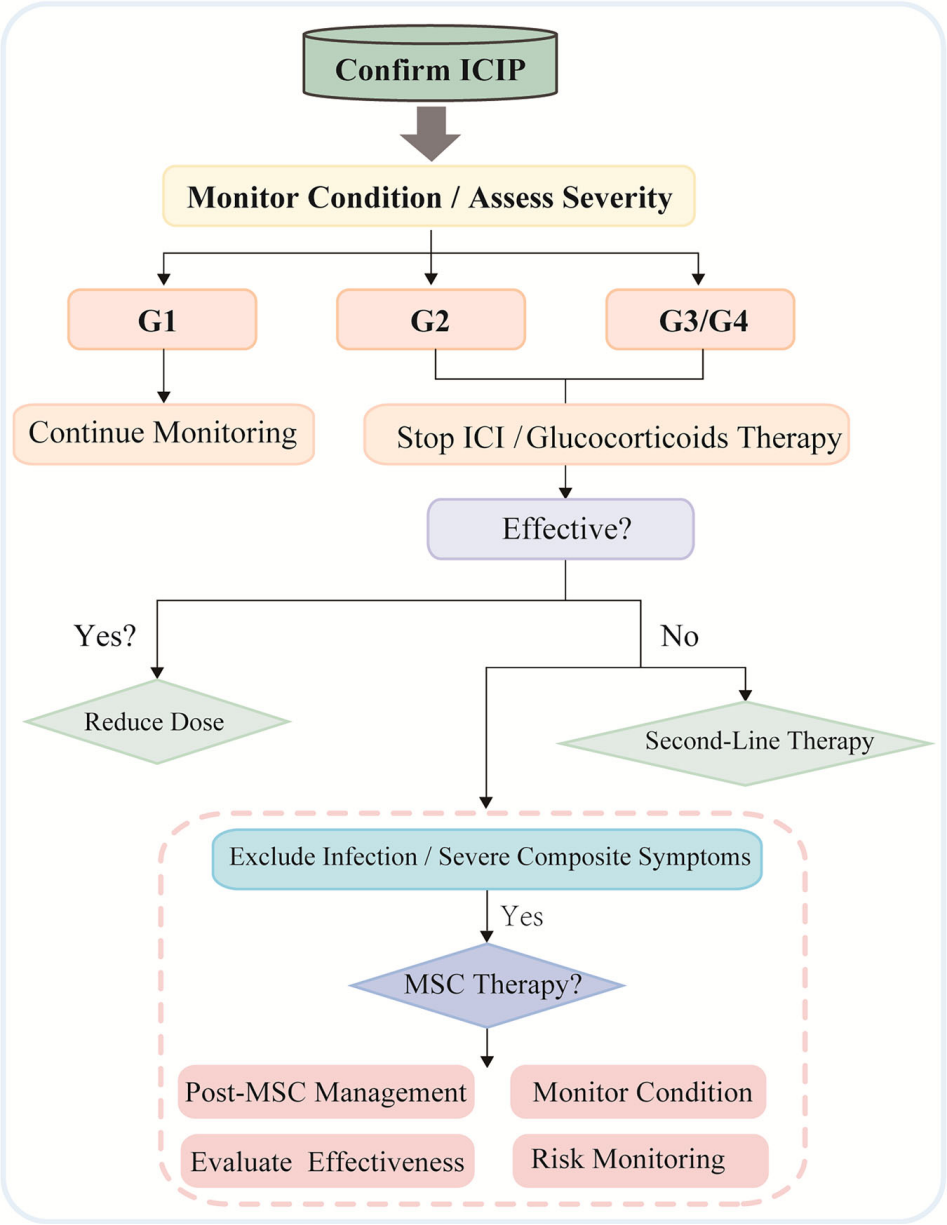
Future prospects of MSC therapy in ICIP Management. After confirming ICIP, monitoring and assessment are conducted based on different severity levels. For high-grade ICIP, whether the effect is significant is evaluated after GC therapy. If the response is inadequate, second-line therapy may be considered. Following the exclusion of infection or severe composite symptoms, the potential for future treatment with MSCs can be explored. ICIP immune checkpoint inhibitor-related pneumonitis, MSCs mesenchymal stem cells, GC glucocorticoids.

The advent of ICIs has opened a new era of cancer immunotherapy, offering hope to patients with cancer. However, as these inhibitors become more widely used in clinical practice, irAEs have increasingly surfaced. ICIP is the most common cause of death among irAEs and is an important cause of forced interruption of immunotherapy. In this article, we explore the potential of stem cell therapy for ICIP and its underlying mechanisms, providing important references for future clinical applications and basic research. However, the treatment options for ICIP remain relatively limited, especially in populations with poor control over corticosteroids. MSC therapy has demonstrated therapeutic value in various diseases, such as acute lung injury and irAEs. Therefore, we propose stem cells as a potential option for ICIP treatment, providing references for the future management of ICIP (Fig. 3).

Notably, the selection of MSCs for treatment also requires attention when addressing ICIP. Bone marrow-derived MSCs (BM-MSCs), adipose-derived MSCs (AD-MSCs), and UC-MSCs are commonly used MSCs; however, they exhibit some differences in proliferation, differentiation, and immunomodulation, which directly contribute to uncertainties in their clinical efficacy. BM-MSCs, known for their strong immunomodulatory and tissue repair capabilities, were among the earliest to be studied. However, their collection process is relatively invasive, and their therapeutic efficacy may be influenced by factors such as donor age and health status [132–134]. UC-MSCs possess notable differentiation potential and significant proliferative ability, and their collection process is less invasive, drawing increasing attention [135–137]. A recent study evaluated the therapeutic potential of BM-MSCs, AD-MSCs, and UC-MSCs in acute respiratory distress syndrome. The results show that UC-MSCs were superior compared to other types of MSCs in regulating immunity, alleviating epithelial/endothelial damage, and reducing lung inflammation [138]. Exosomes derived from AD-MSCs also exhibit lung-protective effects [139].

Challenges for MSCs in clinical applications include the complexity of the application phase. The therapeutic factors released by MSCs are highly dependent on specific conditions in the immune microenvironment, such as the inflammatory state, hypoxic conditions, and extracellular matrix components [140]. This reliance on the microenvironment results in significant variability in the type and amount of factors released by MSCs, which can substantially affect the predictability and stability of therapeutic outcomes. Therefore, in ICIP treatment, carefully considering the impact of the microenvironment on MSCs function is crucial to optimize therapeutic strategies and reduce the uncertainty of potential treatment risks. Engineered stem cells and stem cell-derived exosomes provide new insights for addressing this challenge. Recently, extracorporeal photopheresis (ECP), an immunomodulatory treatment method that involves the extraction, processing, and reinfusion of a patient’s white blood cells, achieved an overall response rate of 92% for treating irAEs. In addition, following ECP treatment, all patients reduced their GC dosage without compromising antitumor immunity [141]. This finding offers valuable insights into cellular therapies that address ICIP.

ICIP, as a serious immune-related adverse reaction, warrants attention from clinicians. MSCs may represent a promising approach for the future treatment of ICIP. However, further studies are required to comprehensively evaluate its efficacy and safety, determine the optimal sources and dosages of MSCs, and standardize treatment protocols. Establishing animal models and conducting clinical trials will be essential to advance this therapeutic approach.
